# 4-Methyl-5-(4-nitro­benzyl­idene)-2-oxo-2,5-di­hydro-1*H*-pyrrole-3-carbo­nitrile

**DOI:** 10.1107/S1600536813017066

**Published:** 2013-06-26

**Authors:** Graeme J. Gainsford, M. Delower. H. Bhuiyan, Andrew J. Kay

**Affiliations:** a Callaghan Innovation Research Limited, PO Box 31-310, Lower Hutt, New Zealand

## Abstract

Mol­ecules of the potential non-linear optical title compound, C_13_H_9_N_3_O_3_, form dimeric stacks of mol­ecules along the *a* axis cross-linked around inversion centers by N—H⋯O hydrogen bonds and weak (phen­yl)C—H⋯O inter­molecular inter­actions, forming a ‘collaboration’ of *R*_2_^2^(8) and *R*_2_^2^(16) ring motifs. The mol­ecules are then further linked by weak C—H⋯O and C—H⋯N inter­actions into sheets parallel to (121).

## Related literature

For hydrogen-bonding motifs, see: Bernstein *et al.* (1995 ▶). For chemical synthesis literature, see: Shrestha-Dawadi & Lugtenburg (2007 ▶). For background literature, see: Bert *et al.* (2006 ▶); Colin *et al.* (2002 ▶); Hasan *et al.* (2012 ▶); Stephen *et al.* (2011 ▶); Tarek *et al.* (2013 ▶). For a description of the Cambridge Structural Database, see: Allen (2002 ▶).
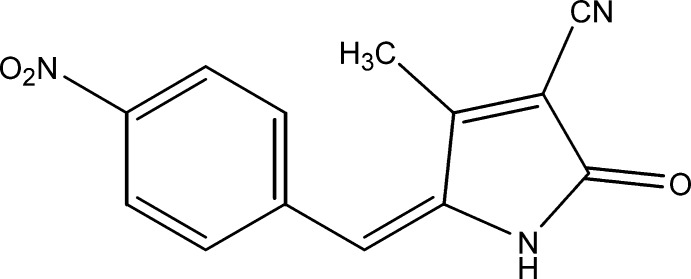


## Experimental

### 

#### Crystal data

C_13_H_9_N_3_O_3_
*M* *_r_* = 255.23
Monoclinic, 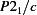

*a* = 3.7456 (2) Å
*b* = 14.9193 (9) Å
*c* = 21.6077 (17) Å
β = 92.273 (7)°
*V* = 1206.52 (14) Å^3^
*Z* = 4
Cu *K*α radiation
μ = 0.86 mm^−1^
*T* = 120 K
0.44 × 0.05 × 0.03 mm

#### Data collection

Agilent SuperNova (Dual, Cu at zero, Atlas) diffractometer
Absorption correction: multi-scan (*CrysAlis PRO*; Agilent, 2011 ▶) *T*_min_ = 0.633, *T*_max_ = 1.000
7096 measured reflections
2283 independent reflections
1941 reflections with *I* > 2σ(*I*)
*R*_int_ = 0.042

#### Refinement

*R*[*F* ^2^ > 2σ(*F* ^2^)] = 0.048
*wR*(*F* ^2^) = 0.139
*S* = 1.05
2283 reflections
173 parameters
H-atom parameters constrained
Δρ_max_ = 0.25 e Å^−3^
Δρ_min_ = −0.25 e Å^−3^

### 

Data collection: *CrysAlis PRO* (Agilent, 2011 ▶); cell refinement: *CrysAlis PRO*; data reduction: *CrysAlis PRO*; program(s) used to solve structure: *SHELXS97* (Sheldrick, 2008 ▶); program(s) used to refine structure: *SHELXL2012* (Sheldrick, 2008 ▶); molecular graphics: *ORTEP-3 for Windows* (Farrugia, 2012 ▶) and *Mercury* (Macrae *et al.*, 2008 ▶); software used to prepare material for publication: *SHELXL97* and *PLATON* (Spek, 2009 ▶).

## Supplementary Material

Crystal structure: contains datablock(s) global, I. DOI: 10.1107/S1600536813017066/jj2169sup1.cif

Structure factors: contains datablock(s) I. DOI: 10.1107/S1600536813017066/jj2169Isup2.hkl

Supplementary material file. DOI: 10.1107/S1600536813017066/jj2169Isup3.cml

Additional supplementary materials:  crystallographic information; 3D view; checkCIF report

## Figures and Tables

**Table 1:** Hydrogen-bond geometry (Å, °)

*D*—H⋯*A*	*D*—H	H⋯*A*	*D*⋯*A*	*D*—H⋯*A*
N1—H1⋯O1^i^	0.88	1.98	2.8256 (18)	159
C9—H9⋯O1^ii^	0.95	2.43	3.336 (2)	159
C12—H12⋯N2^iii^	0.95	2.59	3.374 (3)	141
C13—H13⋯O2^iv^	0.95	2.58	3.372 (2)	141

Symmetry codes: (i) 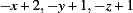; (ii) 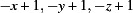; (iii) 
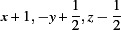
; (iv) 
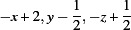
.

**Fig. 1.**
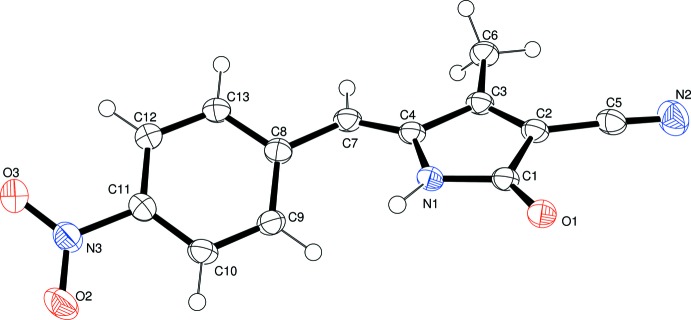
Asymmetric unit atoms for (I) with ellipsoid probability of 50% for non-hydrogen atoms.

**Fig. 2.**
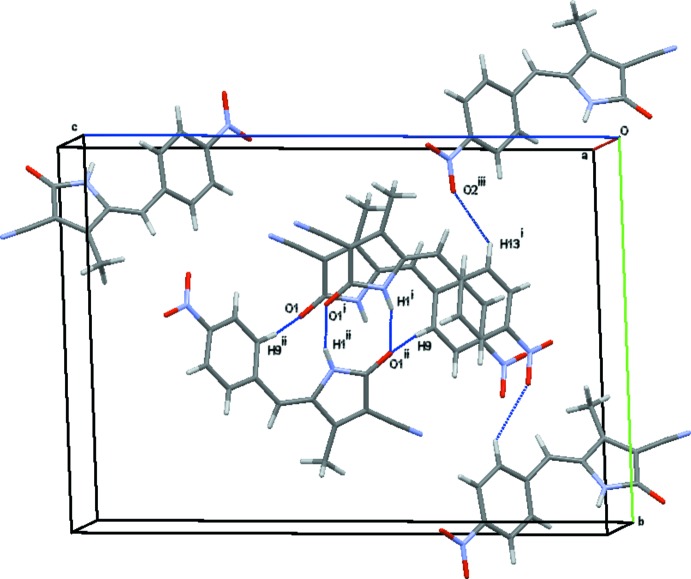
Packing diagram of (I) viewed along the a axis. Intermolecular contacts are shown as blue dotted lines. Symmetry: (i) -1 + x, y, z (ii) 1 - x, 1 - y, 1 - z (iii) 1 - x, -1/2 + y, 1/2 - z.

## supplementary crystallographic information

### Comment

Oxopyrroles and their analogues are the key intermediate for many biologically active compounds (Shrestha-Dawadi & Lugtenburg, 2007) and pigments (Colin *et al.*, 2002). Many studies have been dedicated to the synthesis and spectroscopic characterization of oxopyrroles (Tarek *et al.*, 2013; Hasan *et al.* 2012; Bert *et al.*, 2006) to improve their features. Structural modification has been carried out on the oxypyrrole ring through the introduction of a thiophene ring (Stephen *et al.*, 2011) as an efficient donor and as a precursor for use in organic solar cells. Structural modification *via* the incorporation of an electron-withdrawing group has not been reported.

As a part of our efforts to develop donor-π-acceptor molecules for non-linear optical devices, we have synthesized the title compound in which the oxopyrrole nitrile analogue acts as donor and the nitro group is the acceptor linked by a phenyl-methylene bridge. The molecule crystallizes with one independent molecule in the asymmetric unit (Fig. 1). The 1*H*-pyrole ring is planar with maximum deviation out of plane of 0.018 (2) Å for C2; it makes an angle of 33.99 (9)° with the planar phenyl ring (C8–C13). The nitro group is further twisted by 5.24 (10)° from the latter ring in response to a hydrogen bond interaction with O2. There are few related structures reported and none with linking 5- and 6-membered rings (Allen, 2002; CSD Version 5.34, with Nov 2012 updates).

The molecules form dimers utilizing N—H···O hydrogen bonds about inversion centers of symmetry, packing into approximate planes parallel to the (1,-2,0) plane (Fig. 2). This interaction is further stabilized by weak phenyl(C9)–H ···O1 intermolecular interactions between the adjacent dimers, producing an overall packing "collaboration" of *R*_2_^2^(8) and *R*_2_^2^(16) ring motifs (Bernstein *et al.*, 1995). Other three-dimensional cross-links are provided by chain interactions (not shown in Fig. 2) with weak phenylC–H···O and phenylC–H···N intermolecular contacts (Table 1), linling the molecules into sheets parallel to the (121) plane. .

### Experimental

A mixture of 3-cyano-4-methyl-3-pyrrolin-2-one (Shrestha-Dawadi & Lugtenburg, 2007) (1.0 g), 4-nitro benzaldehyde (1.6 g), sodium acetate (1.8 g) and acetic acid (50 ml) was refluxed for 3 h. under an inert atmosphere. The mixture was stirred overnight, and cooled to 0° C. The resultant precipitate was collected by filtration and washed with cold hexanes to yield the pure chromophore 4-methyl-5-(4-nitro-benzylidene)-2-oxo-2,5-dihydro-1*H*-pyrrole- 3-carbonitrile (1.2 g, 57%) as a yellow solid. Crystals were grown by slow evaporation of an acetone solution. m.p. 277–80° C.

### Refinement

Eight high angle outlier reflection identified by large (F_c_^2^-F_o_^2^)/σ(F_o_^2^) ratios (>4) were OMITted from the dataset.

The methyl H atoms were constrained to an ideal geometry (C—H = 0.98 Å) with *U*_iso_(H) = 1.5*U*_eq_(C), but were allowed to rotate freely about the adjacent C—C bond. All other H atoms were placed in geometrically idealized positions and constrained to ride on their parent atoms with C—H distances of 0.95 Å and N1—H 0 0.88 Å and with *U*_iso_(H) = 1.2*U*_eq_(C,*N*).

### Crystal data

**Table:**

C_13_H_9_N_3_O_3_	*Z* = 4
*M**_r_* = 255.23	*F*(000) = 528
Monoclinic, *P*2_1_/*c*	*D*_x_ = 1.405 Mg m^−^^3^
Hall symbol: -P 2ybc	Cu *K*α radiation, λ = 1.54184 Å
*a* = 3.7456 (2) Å	µ = 0.86 mm^−^^1^
*b* = 14.9193 (9) Å	*T* = 120 K
*c* = 21.6077 (17) Å	Needle, yellow
β = 92.273 (7)°	0.44 × 0.05 × 0.03 mm
*V* = 1206.52 (14) Å^3^	

### Data collection

**Table:**

Agilent SuperNova (Dual, Cu at zero, Atlas) diffractometer	2283 independent reflections
Radiation source: fine-focus sealed tube	1941 reflections with *I* > 2σ(*I*)
Graphite monochromator	*R*_int_ = 0.042
Detector resolution: 10.6501 pixels mm^-1^	θ_max_ = 70.0°, θ_min_ = 3.6°
ω scans	*h* = −4→4
Absorption correction: multi-scan (*CrysAlis PRO*; Agilent, 2011)	*k* = −17→18
*T*_min_ = 0.633, *T*_max_ = 1.000	*l* = −26→18
7096 measured reflections	

### Refinement

**Table:**

Refinement on *F*^2^	Primary atom site location: structure-invariant direct methods
Least-squares matrix: full	Secondary atom site location: difference Fourier map
*R*[*F*^2^ > 2σ(*F*^2^)] = 0.048	Hydrogen site location: inferred from neighbouring sites
*wR*(*F*^2^) = 0.139	H-atom parameters constrained
*S* = 1.05	*w* = 1/[σ^2^(*F*_o_^2^) + (0.0824*P*)^2^ + 0.3085*P*] where *P* = (*F*_o_^2^ + 2*F*_c_^2^)/3
2283 reflections	(Δ/σ)_max_ < 0.001
173 parameters	Δρ_max_ = 0.25 e Å^−^^3^
0 restraints	Δρ_min_ = −0.25 e Å^−^^3^

### Special details

**Table:**

Experimental. Absorption correction: CrysAlisPro, Agilent Technologies, Version 1.171.35.19 (release 27-10-2011 CrysAlis171 .NET) (compiled Oct 27 2011,15:02:11) Empirical absorption correction using spherical harmonics, implemented in SCALE3 ABSPACK scaling algorithm. (MNa)^+^ m/*z* 278.0540; C_13_H_9_N_3_O_3_ requires (MNa)^+^ m/*z* 278.0542). ^1^H NMR (500 MHz, d_6_-DMSO) 2.46 (s, 3H, CH_3_), 6.84 (s, 1H, CH), 7.88 (d, 2H, *J* = 4.9 Hz, ArH), 8.25 (d, 2H, *J* = 4.9 Hz, ArH), 10.91 (s, 1H, NH). ^13^C NMR (75 MHz, d_6_ –DMSO) 12.3, 105.9, 112.7, 113.4, 123.9, 130.9, 138.2, 139.9, 146.7, 161.7, 166.9.
Geometry. All e.s.d.'s (except the e.s.d. in the dihedral angle between two l.s. planes) are estimated using the full covariance matrix. The cell e.s.d.'s are taken into account individually in the estimation of e.s.d.'s in distances, angles and torsion angles; correlations between e.s.d.'s in cell parameters are only used when they are defined by crystal symmetry. An approximate (isotropic) treatment of cell e.s.d.'s is used for estimating e.s.d.'s involving l.s. planes.
Refinement. Refinement of *F*^2^ against ALL reflections. The weighted *R*-factor *wR* and goodness of fit *S* are based on *F*^2^, conventional *R*-factors *R* are based on *F*, with *F* set to zero for negative *F*^2^. The threshold expression of *F*^2^ > σ(*F*^2^) is used only for calculating *R*-factors(gt) *etc*. and is not relevant to the choice of reflections for refinement. *R*-factors based on *F*^2^ are statistically about twice as large as those based on *F*, and *R*- factors based on ALL data will be even larger.

### Fractional atomic coordinates and isotropic or equivalent isotropic displacement parameters (Å^2^)

**Table:**

	*x*	*y*	*z*	*U*_iso_*/*U*_eq_	
O1	0.8538 (3)	0.44087 (8)	0.56486 (6)	0.0277 (3)	
O2	0.9729 (4)	0.64031 (9)	0.18759 (7)	0.0452 (4)	
O3	1.1572 (4)	0.52825 (10)	0.13455 (7)	0.0453 (4)	
N1	0.7576 (4)	0.40243 (9)	0.46186 (7)	0.0243 (3)	
H1	0.8491	0.4502	0.4446	0.029*	
N2	0.3889 (4)	0.24706 (12)	0.63898 (9)	0.0403 (4)	
N3	1.0168 (4)	0.55927 (10)	0.17993 (8)	0.0323 (4)	
C1	0.7387 (4)	0.39026 (11)	0.52376 (8)	0.0236 (4)	
C2	0.5581 (4)	0.30283 (11)	0.53143 (9)	0.0253 (4)	
C3	0.4940 (4)	0.26523 (11)	0.47533 (8)	0.0252 (4)	
C4	0.6126 (4)	0.32905 (11)	0.42854 (8)	0.0246 (4)	
C5	0.4693 (5)	0.27012 (11)	0.59072 (9)	0.0283 (4)	
C6	0.3332 (5)	0.17567 (11)	0.46080 (9)	0.0296 (4)	
H6A	0.1407	0.1825	0.4291	0.044*	
H6B	0.2362	0.1501	0.4984	0.044*	
H6C	0.5171	0.1356	0.4454	0.044*	
C7	0.5892 (4)	0.31806 (11)	0.36711 (8)	0.0264 (4)	
H7	0.4945	0.2624	0.3527	0.032*	
C8	0.6925 (4)	0.38178 (11)	0.31925 (8)	0.0256 (4)	
C9	0.6636 (4)	0.47494 (11)	0.32720 (9)	0.0268 (4)	
H9	0.5724	0.4981	0.3644	0.032*	
C10	0.7663 (5)	0.53323 (11)	0.28161 (9)	0.0283 (4)	
H10	0.7463	0.5962	0.2869	0.034*	
C11	0.8990 (4)	0.49794 (12)	0.22809 (8)	0.0276 (4)	
C12	0.9235 (5)	0.40627 (12)	0.21750 (9)	0.0287 (4)	
H12	1.0116	0.3838	0.1799	0.034*	
C13	0.8157 (5)	0.34892 (12)	0.26339 (8)	0.0285 (4)	
H13	0.8255	0.2860	0.2569	0.034*	

### Atomic displacement parameters (Å^2^)

**Table:**

	*U*^11^	*U*^22^	*U*^33^	*U*^12^	*U*^13^	*U*^23^
O1	0.0363 (6)	0.0202 (6)	0.0267 (7)	−0.0029 (5)	0.0031 (5)	−0.0007 (5)
O2	0.0724 (10)	0.0219 (7)	0.0415 (9)	−0.0063 (6)	0.0049 (7)	0.0049 (6)
O3	0.0579 (9)	0.0402 (8)	0.0392 (9)	0.0065 (7)	0.0202 (7)	0.0088 (7)
N1	0.0314 (7)	0.0158 (7)	0.0258 (8)	−0.0023 (5)	0.0032 (6)	0.0007 (6)
N2	0.0449 (9)	0.0378 (9)	0.0387 (11)	−0.0049 (7)	0.0067 (8)	0.0095 (8)
N3	0.0379 (8)	0.0273 (8)	0.0315 (9)	−0.0017 (6)	0.0015 (7)	0.0049 (7)
C1	0.0264 (7)	0.0178 (8)	0.0268 (9)	0.0020 (6)	0.0036 (6)	0.0007 (7)
C2	0.0278 (8)	0.0184 (8)	0.0300 (10)	0.0014 (6)	0.0054 (7)	0.0025 (7)
C3	0.0268 (8)	0.0179 (8)	0.0313 (10)	0.0005 (6)	0.0048 (7)	0.0010 (7)
C4	0.0283 (8)	0.0168 (8)	0.0289 (9)	0.0012 (6)	0.0045 (7)	0.0001 (7)
C5	0.0311 (8)	0.0196 (8)	0.0342 (11)	−0.0010 (6)	0.0023 (7)	0.0024 (7)
C6	0.0348 (8)	0.0188 (8)	0.0356 (10)	−0.0040 (7)	0.0060 (7)	−0.0009 (7)
C7	0.0296 (8)	0.0179 (8)	0.0320 (10)	−0.0005 (6)	0.0041 (7)	−0.0014 (7)
C8	0.0256 (7)	0.0230 (8)	0.0280 (10)	−0.0008 (6)	0.0003 (7)	−0.0004 (7)
C9	0.0285 (8)	0.0231 (9)	0.0290 (10)	0.0015 (6)	0.0038 (7)	−0.0033 (7)
C10	0.0332 (8)	0.0197 (8)	0.0318 (10)	0.0006 (7)	0.0000 (7)	−0.0001 (7)
C11	0.0289 (8)	0.0253 (9)	0.0285 (10)	−0.0012 (7)	0.0014 (7)	0.0035 (8)
C12	0.0336 (8)	0.0256 (9)	0.0272 (10)	0.0031 (7)	0.0043 (7)	−0.0007 (7)
C13	0.0365 (9)	0.0206 (8)	0.0287 (10)	0.0018 (7)	0.0027 (7)	−0.0019 (7)

### Geometric parameters (Å, º)

**Table:**

O1—C1	1.231 (2)	C6—H6B	0.9800
O2—N3	1.232 (2)	C6—H6C	0.9800
O3—N3	1.222 (2)	C7—C8	1.468 (2)
N1—C1	1.354 (2)	C7—H7	0.9500
N1—C4	1.407 (2)	C8—C13	1.398 (2)
N1—H1	0.8800	C8—C9	1.405 (2)
N2—C5	1.149 (3)	C9—C10	1.380 (2)
N3—C11	1.467 (2)	C9—H9	0.9500
C1—C2	1.482 (2)	C10—C11	1.381 (3)
C2—C3	1.349 (3)	C10—H10	0.9500
C2—C5	1.423 (3)	C11—C12	1.390 (2)
C3—C4	1.470 (2)	C12—C13	1.382 (3)
C3—C6	1.494 (2)	C12—H12	0.9500
C4—C7	1.337 (3)	C13—H13	0.9500
C6—H6A	0.9800		
			
C1—N1—C4	111.50 (14)	H6A—C6—H6C	109.5
C1—N1—H1	124.3	H6B—C6—H6C	109.5
C4—N1—H1	124.3	C4—C7—C8	127.68 (16)
O3—N3—O2	122.93 (16)	C4—C7—H7	116.2
O3—N3—C11	118.96 (15)	C8—C7—H7	116.2
O2—N3—C11	118.11 (16)	C13—C8—C9	118.82 (16)
O1—C1—N1	126.85 (15)	C13—C8—C7	119.10 (15)
O1—C1—C2	127.44 (16)	C9—C8—C7	122.06 (16)
N1—C1—C2	105.70 (15)	C10—C9—C8	120.73 (16)
C3—C2—C5	128.83 (16)	C10—C9—H9	119.6
C3—C2—C1	109.36 (15)	C8—C9—H9	119.6
C5—C2—C1	121.80 (16)	C9—C10—C11	118.51 (16)
C2—C3—C4	107.50 (15)	C9—C10—H10	120.7
C2—C3—C6	128.09 (16)	C11—C10—H10	120.7
C4—C3—C6	124.41 (16)	C10—C11—C12	122.76 (16)
C7—C4—N1	127.73 (15)	C10—C11—N3	118.99 (16)
C7—C4—C3	126.41 (16)	C12—C11—N3	118.24 (16)
N1—C4—C3	105.85 (15)	C13—C12—C11	117.89 (16)
N2—C5—C2	176.98 (19)	C13—C12—H12	121.1
C3—C6—H6A	109.5	C11—C12—H12	121.1
C3—C6—H6B	109.5	C12—C13—C8	121.21 (16)
H6A—C6—H6B	109.5	C12—C13—H13	119.4
C3—C6—H6C	109.5	C8—C13—H13	119.4
			
C4—N1—C1—O1	177.31 (15)	C3—C4—C7—C8	177.49 (16)
C4—N1—C1—C2	−1.53 (17)	C4—C7—C8—C13	148.90 (18)
O1—C1—C2—C3	−175.91 (16)	C4—C7—C8—C9	−32.6 (3)
N1—C1—C2—C3	2.92 (18)	C13—C8—C9—C10	−2.2 (3)
O1—C1—C2—C5	5.4 (3)	C7—C8—C9—C10	179.34 (16)
N1—C1—C2—C5	−175.75 (15)	C8—C9—C10—C11	−0.2 (3)
C5—C2—C3—C4	175.48 (16)	C9—C10—C11—C12	1.9 (3)
C1—C2—C3—C4	−3.06 (18)	C9—C10—C11—N3	−178.58 (16)
C5—C2—C3—C6	−4.5 (3)	O3—N3—C11—C10	174.92 (17)
C1—C2—C3—C6	176.91 (15)	O2—N3—C11—C10	−4.3 (3)
C1—N1—C4—C7	−179.52 (16)	O3—N3—C11—C12	−5.6 (3)
C1—N1—C4—C3	−0.25 (18)	O2—N3—C11—C12	175.26 (17)
C2—C3—C4—C7	−178.61 (16)	C10—C11—C12—C13	−1.2 (3)
C6—C3—C4—C7	1.4 (3)	N3—C11—C12—C13	179.27 (16)
C2—C3—C4—N1	2.11 (18)	C11—C12—C13—C8	−1.3 (3)
C6—C3—C4—N1	−177.87 (15)	C9—C8—C13—C12	2.9 (3)
N1—C4—C7—C8	−3.4 (3)	C7—C8—C13—C12	−178.56 (16)

### Hydrogen-bond geometry (Å, º)

**Table:**

*D*—H···*A*	*D*—H	H···*A*	*D*···*A*	*D*—H···*A*
N1—H1···O1^i^	0.88	1.98	2.8256 (18)	159
C9—H9···O1^ii^	0.95	2.43	3.336 (2)	159
C12—H12···N2^iii^	0.95	2.59	3.374 (3)	141
C13—H13···O2^iv^	0.95	2.58	3.372 (2)	141

Symmetry codes: (i) −*x*+2, −*y*+1, −*z*+1; (ii) −*x*+1, −*y*+1, −*z*+1; (iii) *x*+1, −*y*+1/2, *z*−1/2; (iv) −*x*+2, *y*−1/2, −*z*+1/2.
